# Insights into mammalian transcription control by systematic analysis of ChIP sequencing data

**DOI:** 10.1186/s12859-018-2377-x

**Published:** 2018-11-20

**Authors:** Guillaume Devailly, Anagha Joshi

**Affiliations:** Division of Developmental Biology, the Roslin Institute, University of Edinburgh, Easter Bush Campus, Midlothian, EH25 9RG UK

**Keywords:** ChIP seq, Transcription control, Transcription factors, Transcriptional regulation, Data integration

## Abstract

**Background:**

Transcription regulation is a major controller of gene expression dynamics during development and disease, where transcription factors (TFs) modulate expression of genes through direct or indirect DNA interaction. ChIP sequencing has become the most widely used technique to get a genome wide view of TF occupancy in a cell type of interest, mainly due to established standard protocols and a rapid decrease in the cost of sequencing. The number of available ChIP sequencing data sets in public domain is therefore ever increasing, including data generated by individual labs together with consortia such as the ENCODE project.

**Results:**

A total of 1735 ChIP-sequencing datasets in mouse and human cell types and tissues were used to perform bioinformatic analyses to unravel diverse features of transcription control. 1- We used the Heat*seq webtool to investigate global relations across the ChIP-seq samples. 2- We demonstrated that factors have a specific genomic location preferences that are, for most factors, conserved across species. 3- Promoter proximal binding of factors was more conserved across cell types while the distal binding sites are more cell type specific. 4- We identified combinations of factors preferentially acting together in a cellular context. 5- Finally, by integrating the data with disease-associated gene loci from GWAS studies, we highlight the value of this data to associate novel regulators to disease.

**Conclusion:**

In summary, we demonstrate how ChIP sequencing data integration and analysis is powerful to get new insights into mammalian transcription control and demonstrate the utility of various bioinformatic tools to generate novel testable hypothesis using this public resource.

## Background

The diversity of mammalian organs and tissues is manifested through differences in the gene expression across cell types with the same DNA sequence. To achieve this, specific sets of genes are activated or silenced during development using instructions which include epigenetic and transcription control mechanisms [1]. Throughout development and differentiation, the fate of each cell type is primarily controlled by gene regulation, where genomic regulatory elements receive and execute transcription signals, dependent on their epigenetic state and chromatin accessibility, controlling the expression of key developmental factors [2]. The chromatin immuno-precipitation followed by high throughput sequencing (ChIP-seq) technology successfully maps the protein-DNA interaction at genomic locations in a cellular context [3, 4]. ChIP-seq has been used for the profiling of histone modifications and binding sites of other proteins. In particular, transcription factors (TF) are key players in the regulation of cell-specific gene expression. ChIP-seq of a TF allows the mapping of target regions in both promoters (the region surrounding the gene start, containing regulatory elements) and at gene-distal regions, including enhancers (regulatory elements located far from the corresponding gene start), and allows the subsequent identification of specific sequence motifs bound by a given TF.

The high throughput sequencing data generation is now no more a barrier, but this data is not yet used to its full potential by analysis and integration. The explosion of data has therefore opened new avenues of research. New methods and tools have been developed to facilitate this data-driven biology. The ENCODE consortium has provided in-depth analyses of the TF ChIP-seq generated [5–10]. Despite this, the data remains globally under-exploited and new analyses are both necessary and feasible. Furthermore, other available ChIP-seq datasets have not been investigated as thoroughly as the ENCODE dataset.

**Fig. 1:**
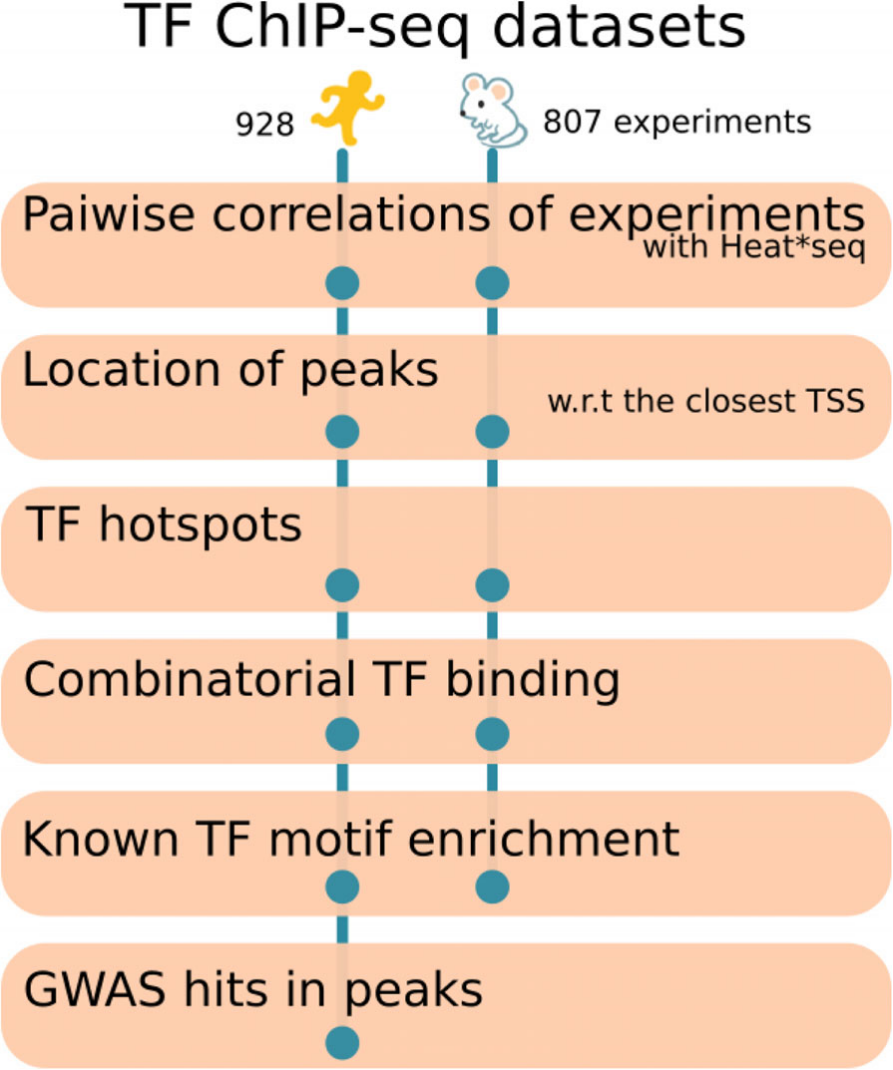
Summary of the analyses performed. Each blue point indicates that the corresponding dataset was used to perform the analysis

We therefore collected ChIP sequencing data from diverse compendia including [11] and consortia [12, 13], resulting in 928 ChIP sequencing samples for transcription related factors in around 100 cell lines and tissues in human and 807 samples in around 50 cell lines and tissues in mouse. We performed a systematic analysis of this data to understand diverse aspects of transcription control across mammalian cell types (Fig. 1). This work is built onto and expands analyses and tools we previously published [14, 15].

## Results and discussion

### HeatChIPSeq for identification of global relationships across experiments

**Fig. 2:**
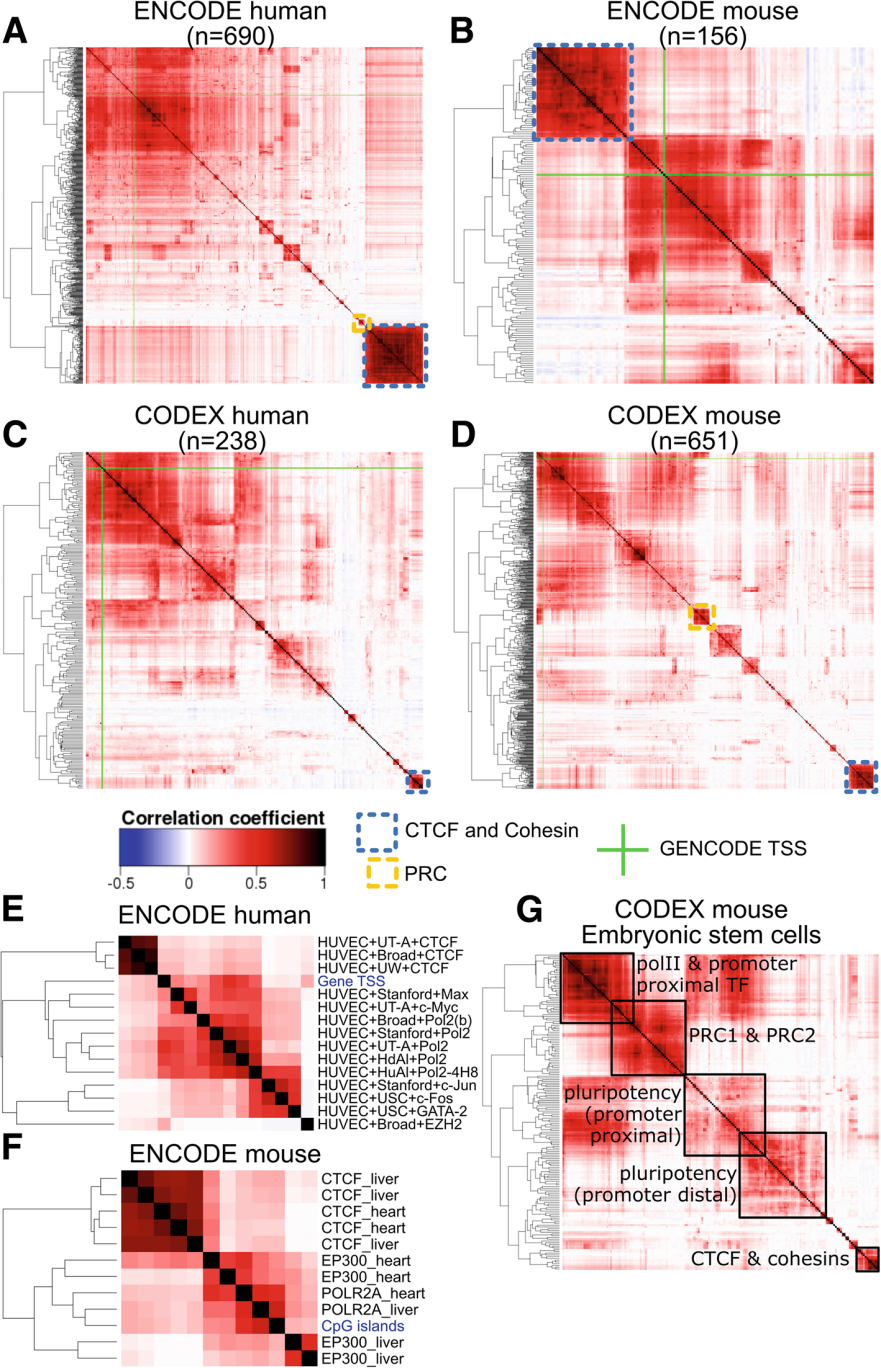
Correlation between ChIP-seq experiments in four datasets. **a-d** Correlation heatmaps of all ChIP-seq experiments included in four datasets: A: ENCODE human, B: ENCODE mouse, C: CODEX human, D: CODEX mouse. Specific clusters are highlighted with annotation after hierarchical clustering of the correlation matrices. TSS locations were added as a track in each heatmaps and are shown in green. **e** A subset of panel A restricted to samples from HUVEC. **f** A subset of the ENCODE mouse dataset showing that while CTCF and RNAPII ChIP-seq clustered together in heart and liver tissues, EP300 ChIP-seq are clustered by tissue. **g** Subset of the CODEX mouse dataset including all ChIP-seq done in mouse embryonic stem cells. Major clusters have been annotated

We analysed four large resources of ChIP-seq datasets, with between 156 and 690 transcription Factor (TF) ChIP-seq experiments from mainly the ENCODE datasets in human (hg19) [12] and mouse (mm10) [13] as well as the CODEX datasets [11] for human (hg19) or mouse (mm10). The number of peaks varied greatly across experiments. For example, a ZNF274 ChIP-seq in HeLa-S3 cell line had only 74 peaks while a CFOS ChIP-seq in MCF10A cell line sample had over 91,000 peaks in the ENCODE human data. We used the HeatChIPseq web tool (Fig. 2), a part of Heat*seq [14] to explore the global relationships across samples by clustering correlation heatmaps from peak overlap data (see methods).

In each datasets we noted similar groupings (Fig. 2). A large fuzzy cluster grouped ChIP-seq experiments with many peaks at promoter regions. This cluster included all RNA polymerase II (RNAPII) ChIP-seq experiments. Gene promoter coordinates were also included in this cluster (Fig. 2a-d, green lines). We noted that many transcription factors clustered according to the cell type rather than the factor. For example, Max and Myc clustered together in NB4 cell line, and also together in K562 cell line in the human ENCODE dataset. The same observation was true in mouse, where Max and Myc clustered together in MEL cell line and also together in CH12.LX cell line in the mouse ENCODE dataset. On the other hand, a very well defined cluster comprised of CTCF and cohesin ChIP-seq experiments from diverse cell types (including RAD21, SMC3, ZNF143) was present in all human and mouse datasets. Other individual clusters had peaks with little or no overlap with promoter or CTCF clusters. Many of these experiments tended to form small clusters of either chromatin regulators or enhancer-binding transcription factors. Globally, but with exceptions, binding profiles of two distinct TFs in the same cell type tended to be more different that binding profiles of the same TF in two different tissues.

The Heat*seq tool allows easy sub-selection of samples in each dataset based on factor or cell type. Figure 2e contains all TF ChIP-seq experiments done in HUVEC (Human Umbilical Vein Endothelial Cells) in the human ENCODE dataset. It illustrates the three main groups of experiments: a tight CTCF cluster, a fuzzy promoter-proximal cluster including RNAPII, MAX and CMYC ChIP-seq experiments, and a cluster with more distal-binding factors such as CJUN, CFOS, and GATA2.

HeatChIPSeq can also be used to explore the fraction of cell-specific binding sites for different factors. Figure 2f includes selected experiments from the mouse ENCODE dataset in liver and heart. CTCF and RNAPII experiments from both tissues clustered together, reflecting mostly shared occupancy between tissues. EP300, on the other hand, formed two separated clusters, one for each tissue, reflecting a higher proportion of tissues specific binding sites.

In Fig. 2g, we selected all experiments from mouse Embryonic stem cells from the mouse CODEX datasets. It allowed characterising further the factors that are neither mainly associated with RNAPII nor CTCF co-factors. We detected a well defined cluster of members from the polycomb complexes PRC1 and PRC2, as well as two clusters composed of pluripotency factors (and other TFs): the first cluster had experiments enriched with promoter-proximal peaks and included factors involved in transcription initiation process or enriched at promoters including Myc and Gata4, while the second cluster, including Sox2, Nanog, Oct4 and Esrrb experiments, had mostly promoter distal peaks. The same factor in the same cell type studied by different labs clustered together with few exceptions such as one Nanog sample in ES cells (GSM1090230) which was closely associated with Kdm4c rather than other Nanog samples.

### Genomic location preference of transcription factors

**Fig. 3:**
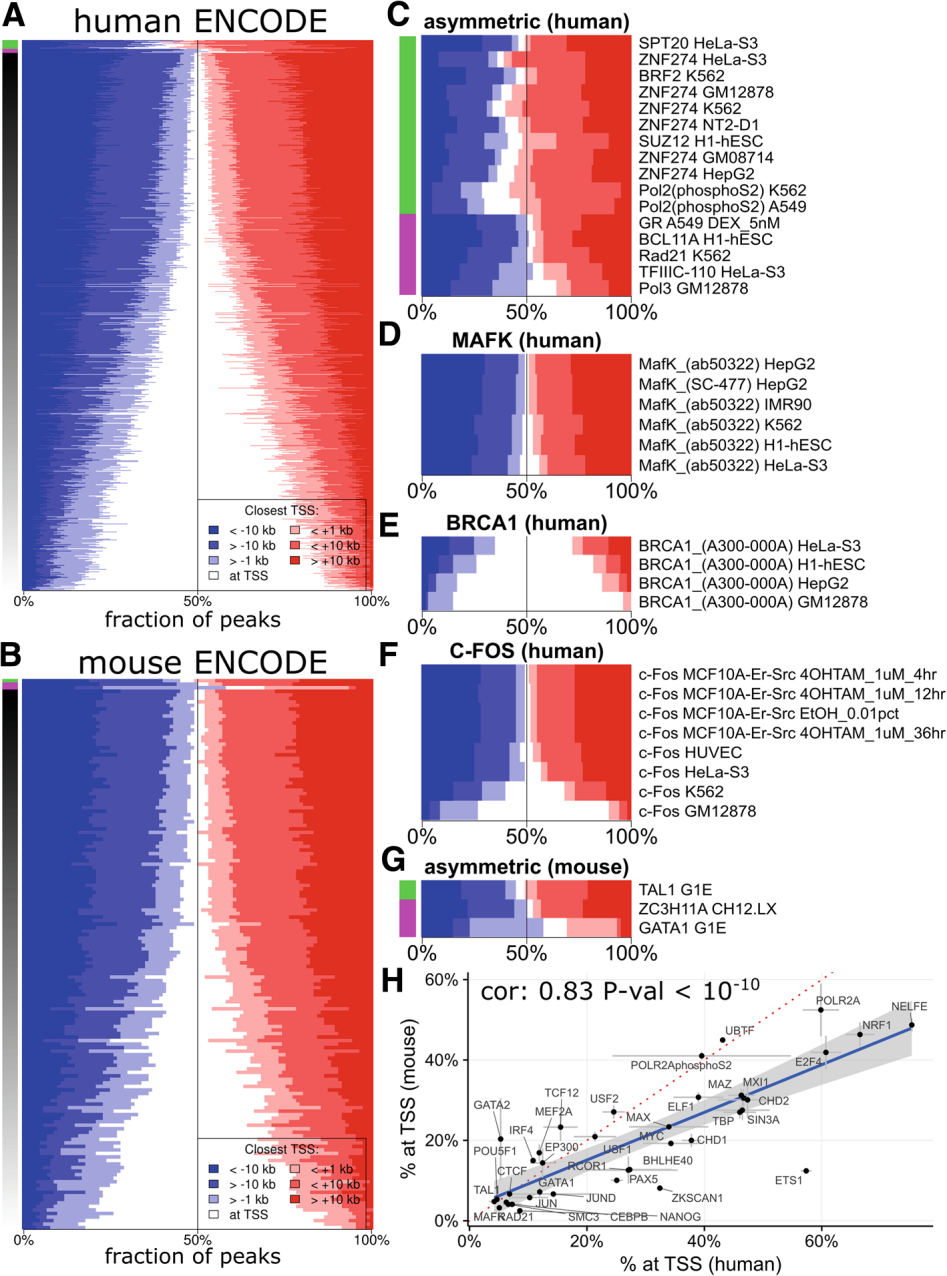
Distribution of peak distances from the nearest TSS. **a** and **b** Stack view of all TF ChIP-seq experiments in human **a** and mouse **b** ENCODE datasets. For each ChIP-seq, fraction of peaks overlapping a TSS (white), upstream (blue) or downstream (red) of the nearest TSS was computed. Experiments were sorted according to the fraction of the peaks overlapping a TSS, to the exclusion of experiments showing more than 50% of peaks upstream (pink side bar) or downstream (green side bar) of the nearest TSS. **c** and **g** A focus on the experiments showing more than 50% of peaks upstream (pink side bar) or downstream (green side bar) of the nearest TSS. **c**: in human. **g** in mouse. **d** MAFK ChIP-seq experiments have few peaks overlapping a TSS. **e** BRCA1 ChIP-seq experiments have most of their peaks overlapping a TSS. **f** C-Fos ChIP-seq experiments have a variable fraction of peaks overlapping a TSS. **h** Fraction of peaks overlapping a TSS was compared between mouse and human for each TF present in both datasets. x-axis: fraction of peaks overlapping a TSS in human. y-axis: fraction of peaks overlapping a TSS in mouse. Grey cross: Range of the Median Absolute Deviation (MAD) of the fraction of peaks overlapping a TSS in cases where several experiments where done for a given TF. Blue line: linear regression. Doted red line: x = y line. cor: Pearson correlation coefficient

The cluster analysis in the previous section indicated that some factors had a majority of their peaks close to gene transcription start sites (TSS) while others were mostly found at promoter-distal regions. We investigated this further by annotating each transcription factor peak (from the human an mouse ENCODE datasets) by its closest GENCODE gene TSS [16, 17] (see methods), and obtained for each factor the fraction of peaks overlapping a TSS, upstream to, or downstream to the nearest TSS (Fig. 3a and b). We divided experiments in three groups: Green labelled experiments (Fig. 3a and b) have more peaks located downstream of a TSS than upstream. This group includes ChIP sequencing experiments for the elongation specific pol II phosphorylated on serine 2, as well as ChIP-seq against ZNF274. ZNF274 (Fig. 3c) has more than 50% of its peaks downstream to the closest TSS, in agreement with Frietze et al. noting that ZNF274 binds the 3’ ends of zinc-finger genes [18]. S2-phosphorylated forms of RNAPII ChIP-seq, a modification reflecting the transcription elongation phase [19], also showed a bias toward more peaks downstream the nearest TSS. On the other hand, experiments indicated with magenta colour (Fig. 3a and b) constituted of experiments with higher fraction of peaks located upstream a TSS than downstream. It included RNA polymerase III and its co-factor TFIIIC (and to a lesser extent RPC155 and BRF1). The third groups contained the majority of experiments with about similar numbers of peaks upstream and downstream of TSS (Fig. 3 and b). Sorting the experiments in the third group by the fraction of peaks overlapping a TSS revealed a continuum, from factors with over 80% of there peaks at a TSS (including most RNAPII or some BRCA1 ChIP-seq experiments, Fig. 3e) to factors with less than 20% of peaks at a TSS (including most CTCF or MAFK ChIP-seq experiments, Fig. 3d).

Most factors showed a conserved fraction of promoter proximal peaks across experiments and or cell types. Nonetheless, we noted a few inconsistent cases. For example, six of eight CFOS ChIP-seq in the human ENCODE datasets had less than 10% of the peaks overlapping a TSS (Fig. 3f), while one experiment had around 30%, and another around 50% of their peaks overlapping a TSS. In the absence of biological replicates, it is difficult to conclude whether these differences reflect a real biological phenomenon or are simply due to technical biases [20].

For each TF, we compared the fraction of peaks overlapping a TSS in mouse and human (Fig. 3h). For most factors, their promoter proximal or distal preference was conserved, with an overall correlation of 0.83 (*P*-value <10^−10^). ETS1 and ZKSCAN1 had a greater fraction of peaks near TSS in human than in mouse, but the lack of replicates hindered assessing whether this is true biological difference or a technical variation. Overall, the linear regression slope was less than one, i.e. most factors had a higher promoter-bound fraction of peaks in human compared to mouse. It is unclear whether this reflects gene annotation differences between the species, gene density differences, or different affinity for promoters in different species.

### Transcription hotspots are enriched strongly both at promoter proximal and distal regions

**Fig. 4:**
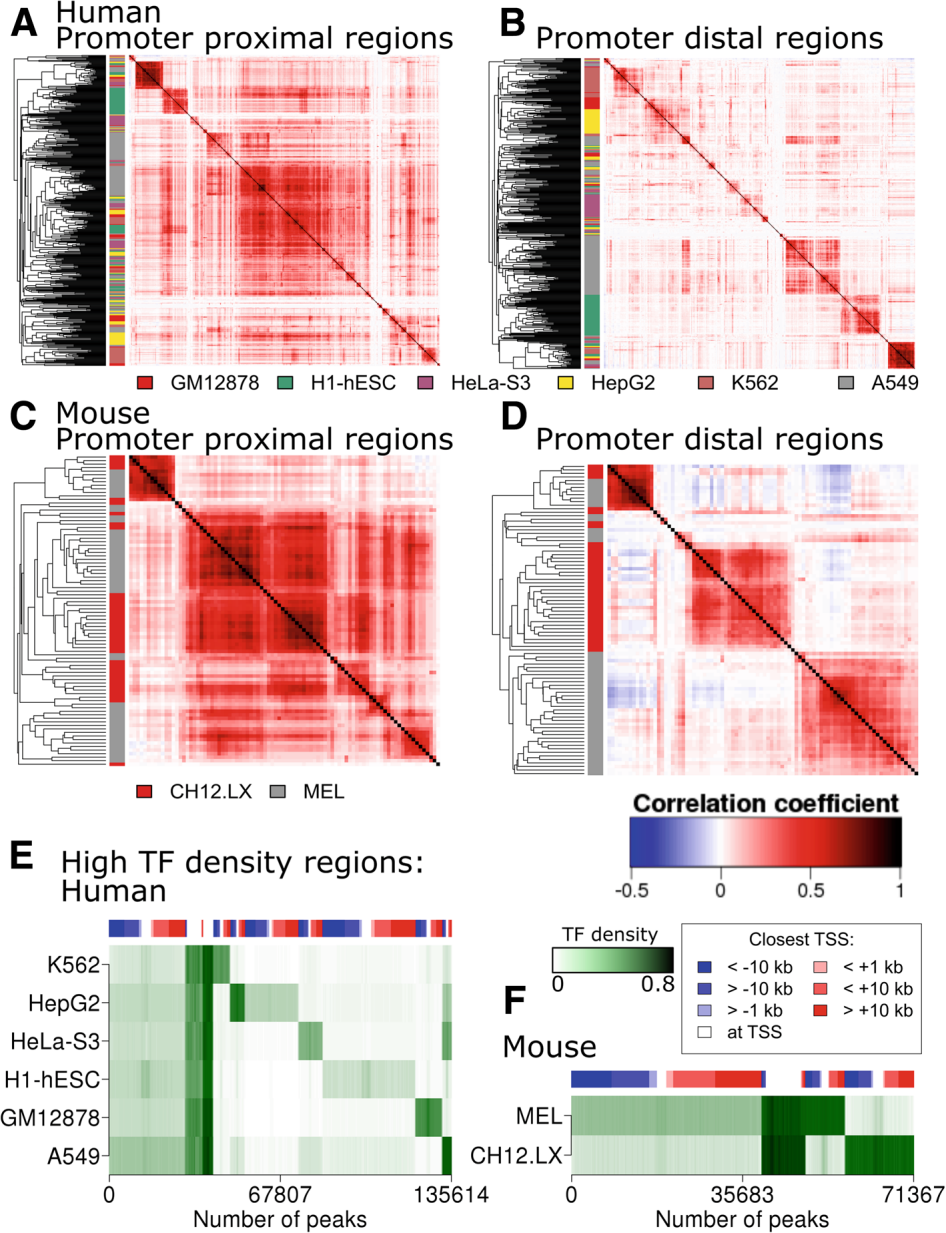
TF peaks farther from TSS are more cell type-specific than peaks overlapping a TSS. **a**-**d** Correlation heatmaps of TF ChIP-seq experiments in human (A and B) or mouse (C and D) datasets from only the peaks at less than 1 kb from the nearest TSS (A and C) or from only the peaks at 10 kb or more of the nearest TSS (upstream or downstream). Colour side bars indicate the cell of origin of the ChIP-seq experiments. Only experiments performed in cell types with more than 30 ChIP-seq experiments are shown. **e** and **f** K-means clustering of highly-bound regions in human **e** and mouse **f**. TF density: proportion of ChIP-seq experiment with a peak at a given location. Top colour bars: proportion of peaks upstream (blue), at (white), or downstream of (red) the nearest TSS. Very highly bound regions in all cells tend to mostly overlap a TSS. Cell specific highly bound regions tends to be more distal. A large lowly TF-bound cluster of genomic regions was removed from the figure for clarity in both human and mouse

We further clustered ChIP-seq experiments using only the promoter proximal or the promoter distal peaks separately, in the six human and the two mouse for cell lines with more than 30 ChIP-seq experiments. In the human ENCODE dataset, six cell lines (A549, GM12878, H1-hESC, HeLa-S3, HepG2, and K562), with a total of 474 experiments were selected (Fig. 4a and b). From the 553,211 regions bound by at least one factor in the 474 experiments, 300,901 were promoter proximal (<1 kb from the nearest TSS), and 144,514 were distal to a TSS (>10 kb). In the mouse ENCODE dataset, two cell lines (CH12 and MEL), with a total of 88 experiments were selected (Fig. 4c and d). From the 221,772 regions bound by at least one factor in the 88 experiments, 33,171 were promoter proximal (<1 kb from the nearest TSS), and 101,150 were distal to a TSS (>10 kb from the nearest TSS). We calculated the Pearson correlation coefficient between each pair of peak lists for the promoter proximal regions (Fig. 4a and c), and for the distal regions (Fig. 4b and d). The hierarchical clustering of the resulting correlation matrix for each of the two sets demonstrated that promoter regions share binding sites for many more factors compared to the distal regions. Indeed, correlation values between experiments were on average higher at promoter-proximal sites than at distal sites. The factors studied in the same cell type clustered together more often at the distal regions than at the promoter regions. The promoter proximal clustering in both human and mouse represented a tight CTCF/SMC3/RAD21 cluster and a large dispersed multi-factor cluster including diverse factors. At distal regions, the CTCF/SMC3/RAD21 cluster remained intact but many other experiments clustered according to their cell line of origin, meaning that TF binding at distal regulatory regions tends to be cell type specific.

Transcription factor hotspots are defined as genomic regions occupied by many factors in a given cellular context. They overlap with regulatory regions that are highly important for cell identity [21]. We characterized transcription hotspots in human and mouse datasets by computing the TF binding density, i.e. the fraction of ChIP-seq experiments in each cell type with a peak in a given genomic region. The clustering of TF density for all genomic regions by k-means clustering (Fig. 4e and f) demonstrated that transcription factors hotspots are promoter proximal and distal. Transcription factor hotspots that are shared by all cell types are mostly found at the promoter regions, while transcription hotspots that are cell type specific are mostly found at non-promoter regions. This is in full agreement with our previous findings.

### Combinatorial control of transcription

Mammalian transcription factors are known to work together by binding at the promoter or enhancer regions to activate or repress downstream target genes [22–24]. Importantly, individual transcription factors known to function combinatorially, i.e. even when expressed at high levels, are not sufficient to activate specific gene expression programs when alone [25]. To unravel the combinatorial control of transcription factors in a cellular context, we grouped factors according to the cell type in mouse and human for cell types with at least 6 factors studied. We then built a M*N matrix of all binding events (peaks) for each cell type where M represents the loci (genomic location) bound by at least one factor in that cell type and N being the number of factors studied by ChIP sequencing in that cell type. For each cell type, there were 2N-1 combinations of binding patterns possible. We evaluated likelihood of frequency of these combinatorial patterns to occur by chance by comparing to 100 random datasets generated such that the total number of binding events for each factor was preserved (i.e. the number of non-zeros in each column of M*N matrix were unaltered during randomizations). This analysis re-discovered the transcription factors of the same family (e.g. SMC family members in murine ES cells, CEBP family members in murine macrophages, Stat family members in murine dendritic cells) known to bind to overlapping genomic locations due to highly similar sequence motifs, or the components of known complexes or well studies interactions (Table 1). CTCF, RAD21 and ZNF143 form a part of cohesin complex and clustered together in the global clustering considering all peaks. Accordingly, they were enriched across multiple cell types. In agreement with the literature, in mouse ES cells, 2350 genomic loci were occupied only by Oct4, Sox2 and Nanog (Bonferroni corrected *P*-value < 10^−256^) [22]. In mouse MEL cells, Gata1 and Scl were co-bound at 1123 (Bonferroni corrected *P*-value < 10^−256^) genomic loci [26]. Interestingly, we identified some novel combinatorial relationships whose functional relevance needs further investigation. For example, in mouse ES cells, 2655 genomic loci were co-occupied only by Suz12 and Sox2 (Bonferroni corrected *P*-value < 10^−256^) and in human GM12878, ETS1 co-occupied 1090 binding sites only with EGR1 (Bonferroni corrected *P*-value < 10^−256^) and 1249 binding sites only with P300 (Bonferroni corrected *P*-value < 10^−256^).

**Table 1:** Top 3 significant associations between factors in mouse and human cell types

Cell type	Combination 1	Combination 2	Combination 3
B cells (M)	E2A, FoxO1, Pax5	E2A, Ebf1, Oct2	Pax5, Smad3, FoxO1A
T cells (M)	Stat3, Stat4, Stat5, Stat6	Stat5a, Stat5b, Stat5	Fli1, Gata3
Dendritic cells (M)	Hif1a, Irf1, Maff, Relb, Stat3, Rel, Irf2Irf4	Hif1a, Irf1, Maff, Relb, Rel, Irf2Irf4	-
Macrophages (M)	CEBPA, CEBPB	CEBPA, CEBPB, PU1, STAT1	CEBPA, CEBPB, PPARG, PU1, STAT1
Erythroid cells (M)	ETO2, GATA1, LDB1, MTGR1, SCL	GATA1, LDB1, MTGR1, SCL	-
MK progenitors (M)	CBFB, GATA1, GATA2, RING1B, RUNX1	CBFB, ETS1, RING1B, RUNX1	-
MEL (M)	JunD, SMC3	GATA1, SCL	CMYC, MAX, MXI1, NELFE, SCL, TBP
ES cells (M)	Suz12, SOX2	E2F1, nMYC	E2F1, KLF4, nMYC
A549 (H)	HDAC6, P300, ELF1, ETS1, GABP	ATF3, BRF1	RNA polII, CTCFL
GM12878 (H)	SAP30, TAF7	STAT5A, BRG1	P300, ETS1
H1-hESC (H)	RAD21, ZNF143	BACH1, MAFK	USF1, USF2
HeLa-S3 (H)	EZH2, RNA polII, SIN3A, CJUN, CMYC	GTF2B, NR2F2	RNAPII, RBBP5
HepG2 (H)	TAF1, TAF7, TEAD4	SAP30, ATF1, ATF3	PU1, STAT5A
K562 (H)	GTF2F1, CTCF	HDAC1, CJUN	E2F6, CTCF

All associations were predicted at very high significance (all *P*-values <1e-256). M - mouse, H - human

Interestingly, there were a number of cases of combinatorial control where the two factors did not share most of the binding sites, therefore did not cluster together in the global clustering tree (Fig. 2) but significantly co-occupied a relatively small but statistically significant number of gene loci. For example, GTF2F1 and CTCF co-bound 2233 genomic regions in human K562 cell line (Bonferroni corrected *P*-value < 10^−256^) which were not occupied by any other factor. Similarly, E2A, FOxO1 and Pax5 co-bound 1087 genomic regions in mouse B cells (Bonferroni corrected *P*-value < 10^−256^) which were not occupied by any other factor studied. In human ES cells, 957 genomic loci were co-occupied only by RAD21 and TEAD4 (Bonferroni corrected *P*-value < 10^−30^). This postulates site specific role for these combinatorial interactions shadowed by the global analysis.

### DNA sequence motif preference of factors

To investigate the sequence motif preference in genome wide biding patterns for each factor defined by ChIP sequencing experiment in a cellular context, we computed the enrichment of known motifs for each sample using HOMER [27]. As expected, for the majority of the experiments, we detected the sequence motif specific to the factor as the top motif enriched for the factor. For example, a CTCF motif was top top motif of all CTCF ChIP sequencing experiments, a Runx1 motif was the top motif for all Runx1 ChIP sequencing experiments, and a GATA motif was enriched as the top motif for all GATA (Gata1, Gata2 or Gata3) ChIP sequencing experiments across all cell types studied in human and mouse. This firstly confirmed the quality of the most of the data in the compendia. This analysis furthermore highlighted known interacting partners of some factors: all LMO2 samples had ETS as the top motif, all MYB samples had RUNX as the top motif and all CBFB samples had CTCF as the top motif.

Tal1/Scl experiments had mostly ETS or RUNX or GATA as the top enriched motif, depending upon the cell type in which the factor was studied in both human and mouse. Similarly, the majority of RNAPII experiments across multiple cell types identified ETS as a top enriched motif, with only a handful of cases with a cell type motifs enriched such as GATA motif enriched in K562 RNAPII sample, or BZIP motif enriched in HeLa-S3 RNAPII sample. In embryonic stem cells (ESCs), OCT4-SOX2-TCF-NANOG motif was enriched for the ChIP sequencing of OCT4 and NANOG as expected; this motif was top enriched motif in SMAD3, P300, BCL11A, HDAC2 and CTBP2 samples as well. Interestingly, CTCF ChIP sequencing in different cell types did not result in enrichment of sequence motifs of cell type specific factors. This demonstrates that CTCF acts mainly as an insulator as well as defining gene regulatory boundaries which are largely independent of cell type.

### Transcription control of disease susceptibility loci

Genetic variations can be one of the causal factors of complex diseases. Genome-wide analysis (GWAS) studies have revealed genetic loci significantly associated with a disease risk, but the majority of the identified loci lie within the non-coding regions, specifically in the regulatory regions defined by chromatin modifications and DNase I hypersensitive sites across cell types [28]. We calculated the overlap of binding sites for each transcription factor in human in each cell type with GWAS high confidence hits and estimated the significance of overlap (whether or not possible simply by random chance) using bonferroni-corrected hypergeometric test. Three ChIP sequencing samples from the ENCODE data showed statistically significant overlap with GWAS disease associated loci. These tree factors, NELFE and HDAC8 in K562 cell line and BRCA1 in GM12878 cell line, with about 2% of the peaks for each factor in GWAS disease associated loci, have a well-studied role associated with cancer. BRCA1 mutations are causal for breast cancer [29]. Accordingly, the majority BRCA1 peaks overlapped with the disease loci in breast cancer. Interestingly, 37 BRCA1 peaks overlapped with disease loci in inflammatory bowel disease. BRCA1 also has a role as an important mediator of innate immunity and BRCA1 gene therapy is known to reduce systemic inflammatory response [30]. We noted that 12 BRCA1 target loci overlapped with genes involved in childhood obesity. This is in agreement with a finding that without BRCA1, muscle cells became diabetic by storing excess fat [31]. Taken together, the analysis of transcription targets using ChIP sequencing together with disease associated loci can identify novel factors controlling the disease phenotype.

## Conclusions

As the next generation sequencing is becoming a preferred tool of the experimental groups world-wide, the genome wide data will be ever increasing in public domain. Understanding the potential of this data for novel discoveries, huge efforts are ongoing in storing and assembling data in public repositories and databases [32–34]. One of the major challenges now is to generate methodologies and pipelines facilitating computational analysis of this large and under-exploited resource. This can be achieved in a plethora of ways, including integrating together with other datasets, to obtain new biological insights. To this end, the ENCODE consortium has taken a big initiative to provide a uniformly processed dataset to the scientific community for computational data integration and analysis [12]. Similarly CODEX database [11] provides a uniformly processed and analysed data from blood and ES cells in human and mouse. The easy accessibility of pre-processed data is a major facilitator for generation of novel biological hypotheses from this resource.

In this paper, we explored multiple resources and tools (Fig. 1), including integration with other data resources such as GWAS catalogue. We demonstrated the use of HeatChIPseq webtool to investigate global relations across ChIP-seq samples. We further analysed the genomic location preference of TFs across species. We showed that transcription hotspots are both promoter proximal and distal. We confirmed that promoter proximal binding of factors (including at transcription hotspots) is more shared across tissues, while the distal binding is more cell type specific. We identified combinations of factors preferentially acting together. Finally, we integrated the data with disease-associated gene loci from GWAS studies to highlight the value of this data to associate novel regulators to disease.

In summary, we demonstrate potential ways to develop new hypotheses about transcription control mechanisms. Importantly, the above results form only the tip of the iceberg of the potential insights from these resources. Our analysis has allowed us to reproduce and expand some observations previously made using these data. This shows that the in depth analysis of such data is still far from complete and must continue. Importantly, the analysis performed in this study can easily be extended to exploit ChIP-seq datasets in other species or in other cellular contexts, and therefore has the potential to significantly advance our understanding of a wide range of both normal and pathological cellular processes.

## Methods

Peaks bed files where downloaded from ENCODE at UCSC (human data, http://hgdownload.cse.ucsc.edu/goldenPath/hg19/encodeDCC/wgEncodeAwgTfbsUniform/) or from the ENCODE data portal (mouse, encodeproject.org) for a total of 690 experiments in human and 156 experiments in mouse. Human and mouse peak files from ChIP-seq experiments were also downloaded from CODEX [11] (codex.stemcells.cam.ac.uk ), for a total of 238 experiments in human and 651 experiments in mouse.

For each of four datasets, all peaks lists were merged using bedtools merge [35] to find non-overlapping peak-containing regions. The merged peaks where then used to create a genomic regions x experiments binary matrix where each matrix cell indicates the presence or absence of a peak at a given region in a given experiment. This binary peak matrix was correlated and clustered, using Euclidean distance of the correlation values and complete hierarchical clustering from the relevant R functions. Clustering branches were re-ordered using the order.optimal function from the cba package [36]. Processed data and detailed dataset processing instructions are available through our web application Heat*seq [14] and the related GitHub repository github.com/gdevailly/HeatStarSeq_gh . The mouse ENCODE dataset was implemented specifically for this study. TSS lists where obtained from GENCODE [16, 17], using the median TSS for genes with several alternative transcription start sites. CpG islands coordinates where obtained as a bed files from the UCSC table browser [37].

Individual peak files and merged peak lists from the human and mouse ENCODE datasets were annotated with the closest GENCODE [16, 17] TSS (v21 for human, after using lift-over to convert it back to hg19, vM14 for mouse) using Bedtools [35] closest command with the -D parameter. Annotated peak lists were used to analyse the distribution of peaks upstream to, at, or downstream to the nearest TSS.

Annotated merged peak lists were used to create binary genomic regions x experiments matrices of only the regions at less than 1 kb from the closest TSS or only the regions at more than 10 kb from the closest TSS. Promoter proximal and promoter distal matrices were then clustered as described before, using only experiments in the 6 cell lines (humans) or the 2 cell lines (mouse) with the most experiments (ENCODE data). The binary matrices were used to compute the TF density of each merged peaks for each cell line, defined as the number of experiment with a peak at a given location divided by the total number of experiments from the cell line in a dataset. TF density matrices of merged peaks x cell line were clustered using k-mean clustering to identify clusters of high TF density that were either ubiquitous or cell-line specific.

From each matrix of non-overlapping regions x experiments (ENCODE data for human and mouse), we computed Transcription Factor (TF) density by dividing the number of factors with a peak in that region by the total number of ChIP sequencing experiments done in that cell line, to obtain numbers between 0 and 1. The promoter proximal regions were defined as within 1kb of GENCODE TSS for both human and mouse, while promoter distal were further than 1kb from the nearest TSS. TF density matrices were then clustered using k-means clustering.

TO study the combinatorial control of transcription factors in a cell type, the cell types with more than 6 factors studied were chosen and over-representation of each combination was calculated using random binding sets and estimating Bonferroni corrected *P*-value. The random binding sets maintained the same number of targets for each TF.

The sequence motif enrichment for each ChIP-seq sample was performed using HOMER [27] with all peak locations as an input. A list of significantly disease associated genomic regions were downloaded from www.ebi.ac.uk/gwas/. P values were calculated using hypergeometric test, and were adjusted using Bonferroni correction. The enriched combinatorial patterns were calculated by generating random binding data keeping the same number of peaks for each factor, and the significance was estimated compared to 100 randomizations.
